# Supplementary figures and images for: Evolutionary rate covariation is pervasive between glycosylation pathways and points to potential disease modifiers
Source: PLoS Genet. 2024 Sep 11;20(9):e1011406. doi: 10.1371/journal.pgen.1011406 (PMC11419382; doi:10.1371/journal.pgen.1011406)

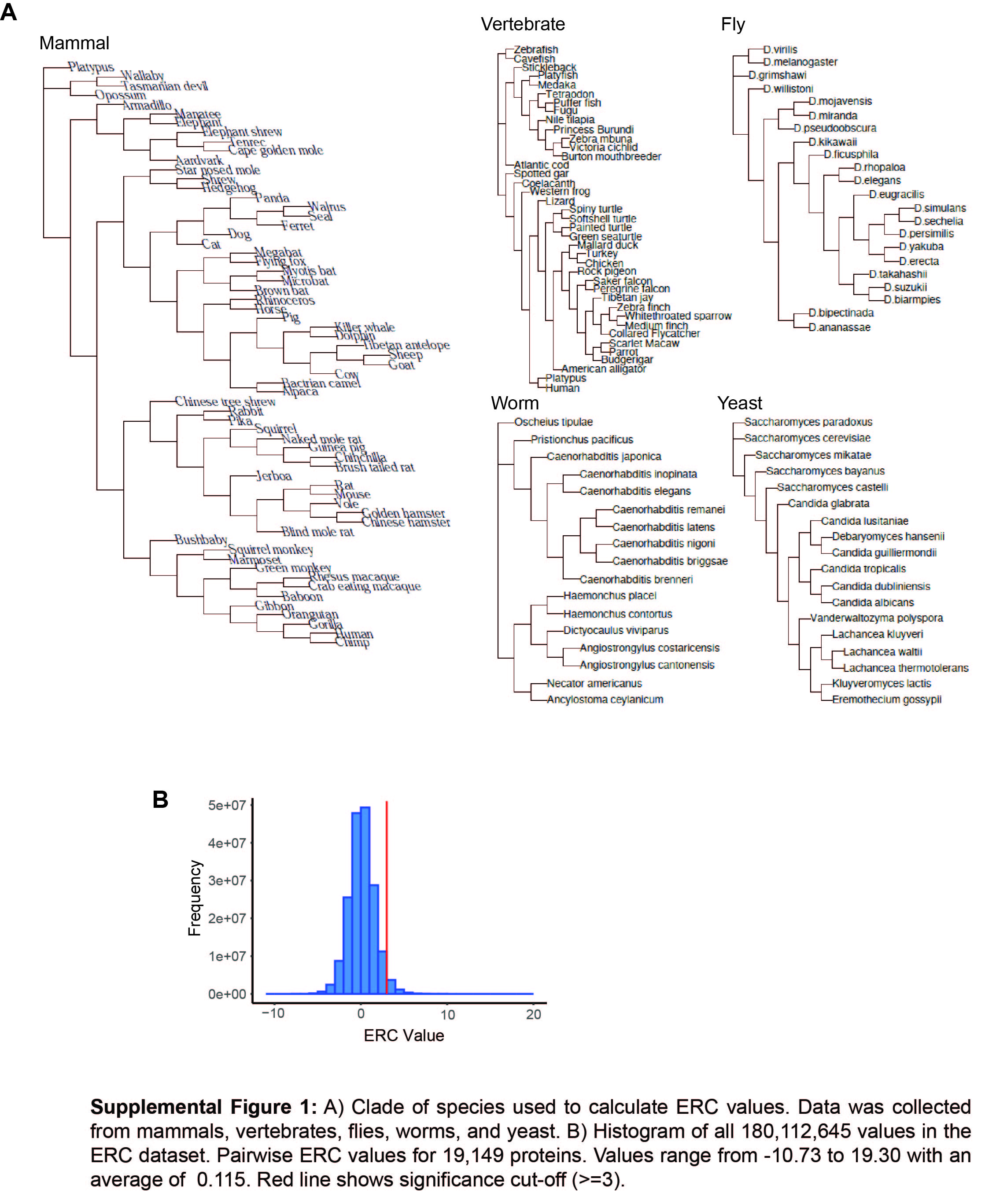

Supplement: S1 Fig — A) Clade of species used to calculate ERC values. Data was collected from mammals, vertebrates, flies, worms, and yeast. B) Histogram of all 180,112,645 values in the ERC dataset. Pairwise ERC values for 19,149 proteins. Values range from -10.73 to 19.30 with an average of 0.115. Red line shows significance cut-off (> = 3). (JPG) [file pgen.1011406.s001.jpg]

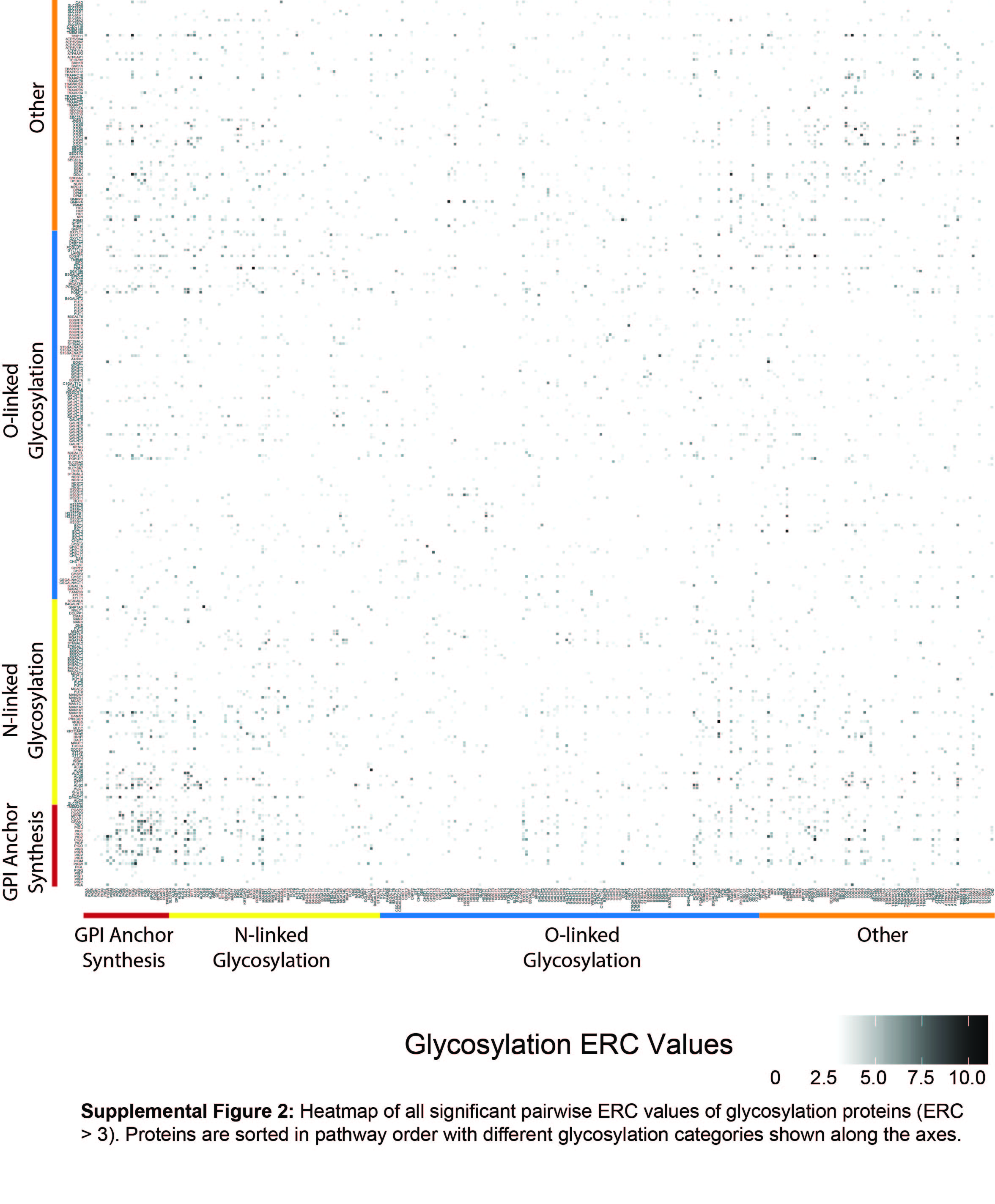

Supplement: S2 Fig — Proteins are sorted in pathway order with different glycosylation categories shown along the axes. (JPG) [file pgen.1011406.s002.jpg]

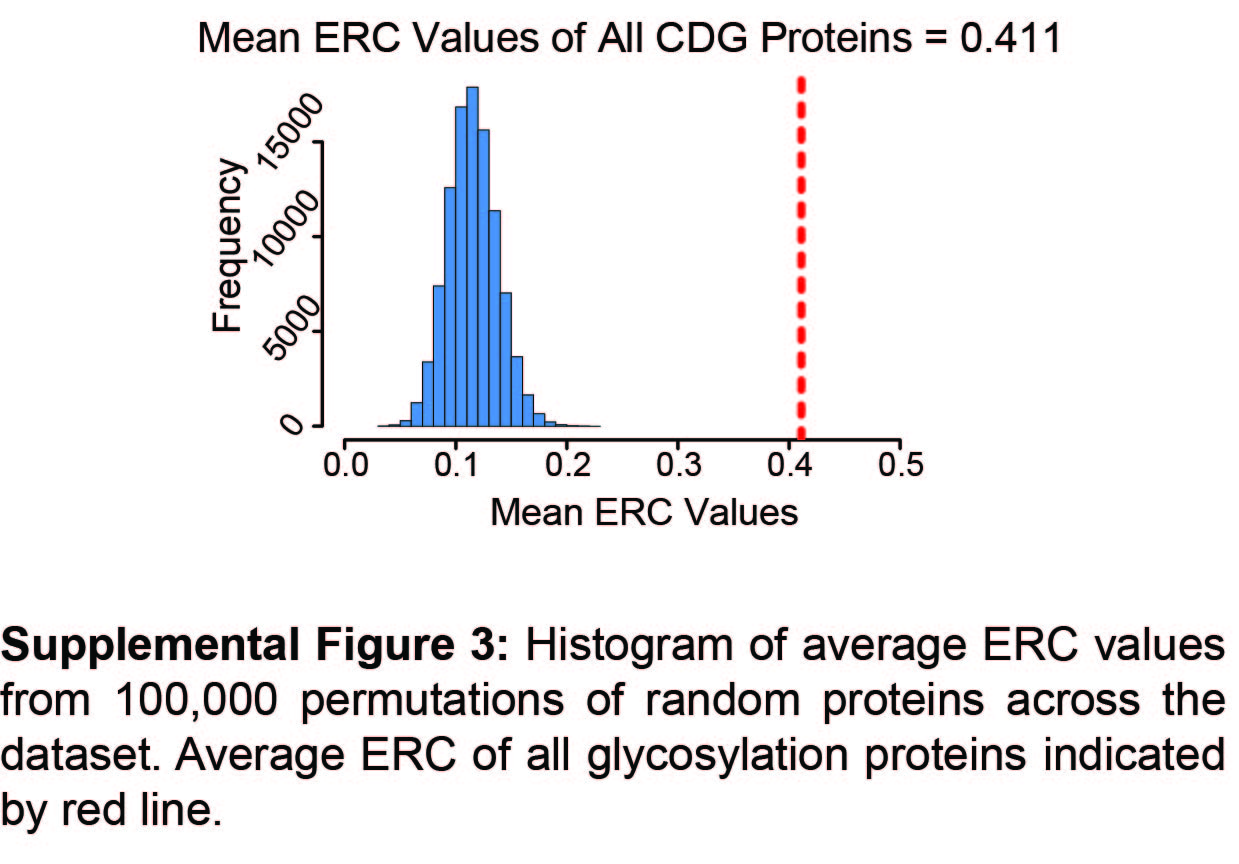

Supplement: S3 Fig — Average ERC of all glycosylation proteins indicated by red line. (JPG) [file pgen.1011406.s003.jpg]

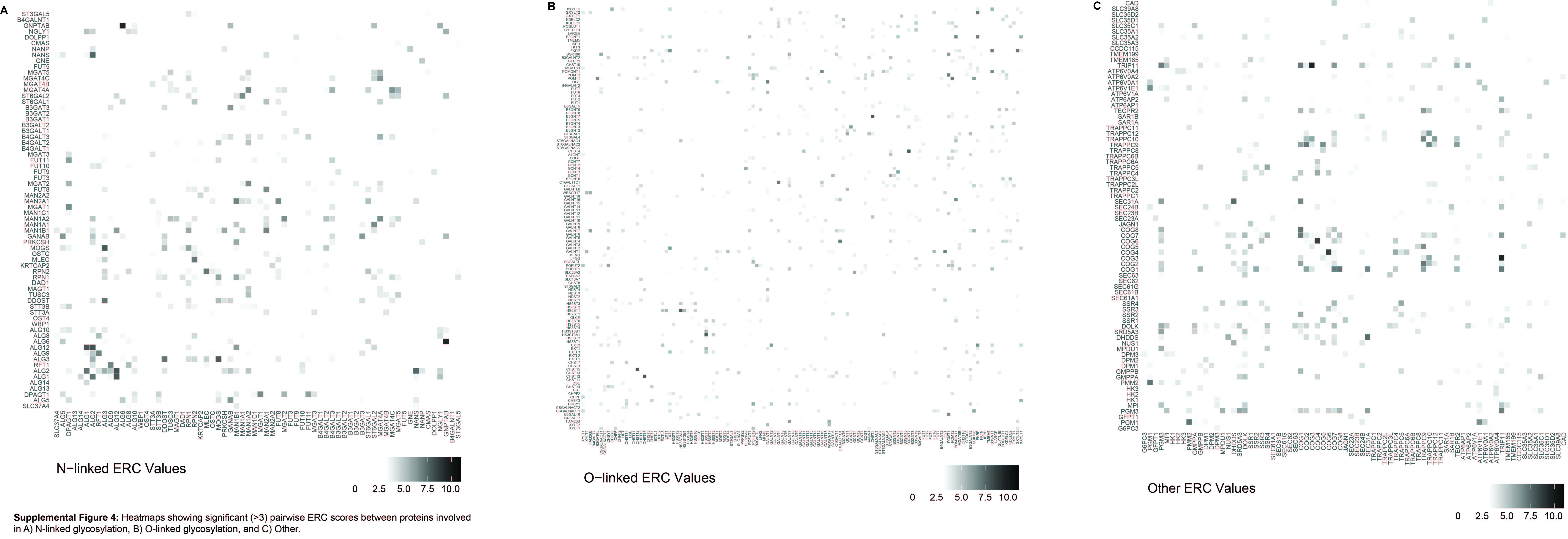

Supplement: S4 Fig — Heatmaps showing significant (>3) pairwise ERC scores between proteins involved in A) N-linked glycosylation, B) O-linked glycosylation, and C) Other. (JPG) [file pgen.1011406.s004.jpg]

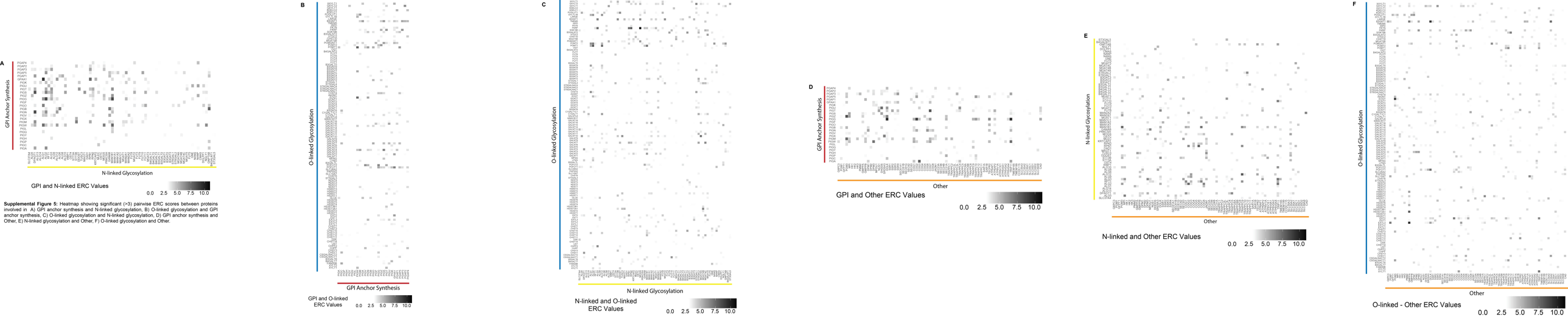

Supplement: S5 Fig — Heatmap showing significant (>3) pairwise ERC scores between proteins involved in A) GPI anchor synthesis and N-linked glycosylation, B) O-linked glycosylation and GPI anchor synthesis, C) O-linked glycosylation and N-linked glycosylation, D) GPI anchor synthesis and Other, E) N-linked glycosylation and Other, F) O-linked glycosylation and Other. (JPG) [file pgen.1011406.s005.jpg]

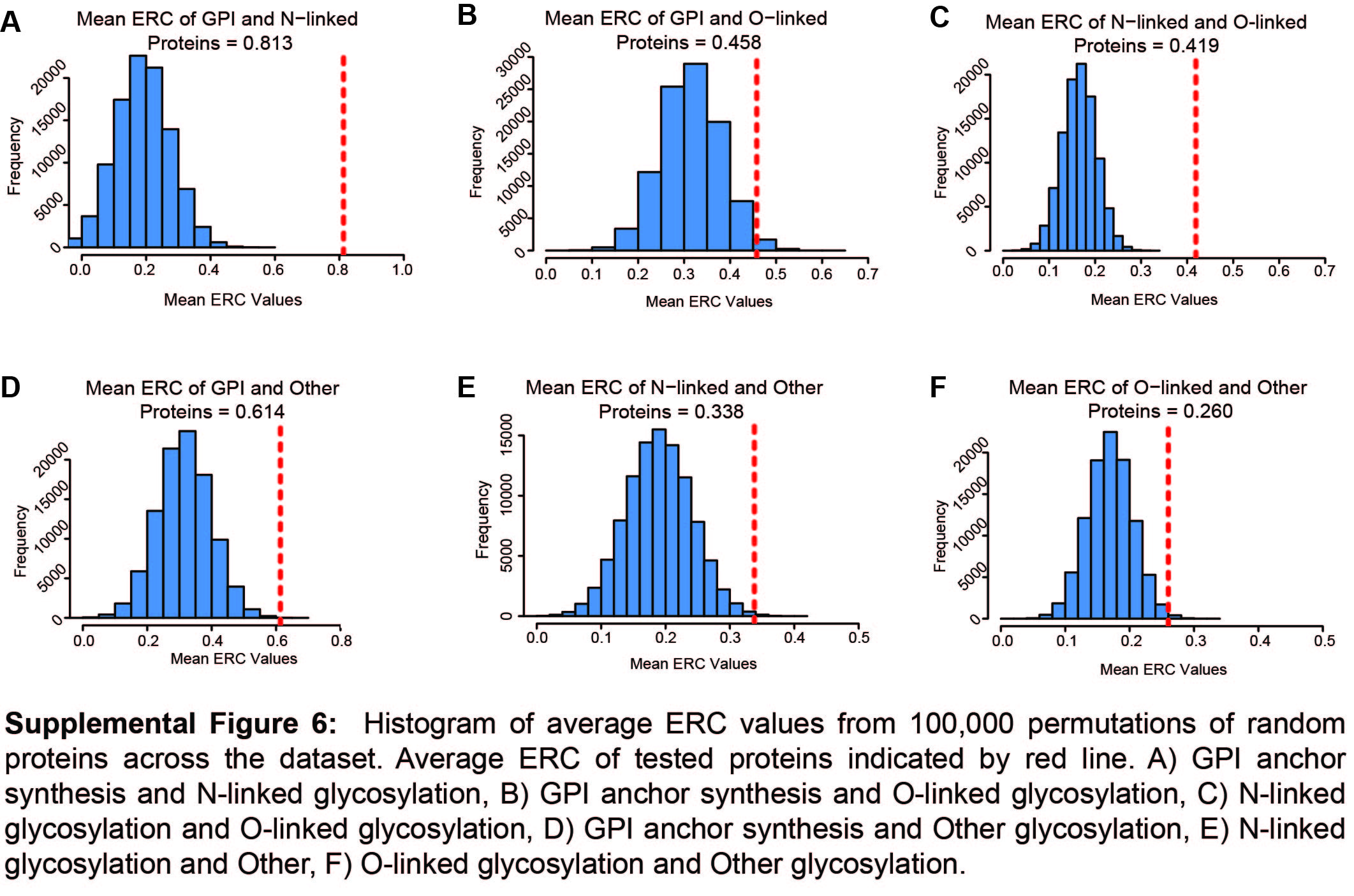

Supplement: S6 Fig — Average ERC of tested proteins indicated by red line. A) GPI anchor synthesis and N-linked glycosylation, B) GPI anchor synthesis and O-linked glycosylation, C) N-linked glycosylation and O-linked glycosylation, D) GPI anchor synthesis and Other glycosylation, E) N-linked glycosylation and Other, F) O-linked glycosylation and Other glycosylation. (JPG) [file pgen.1011406.s006.jpg]

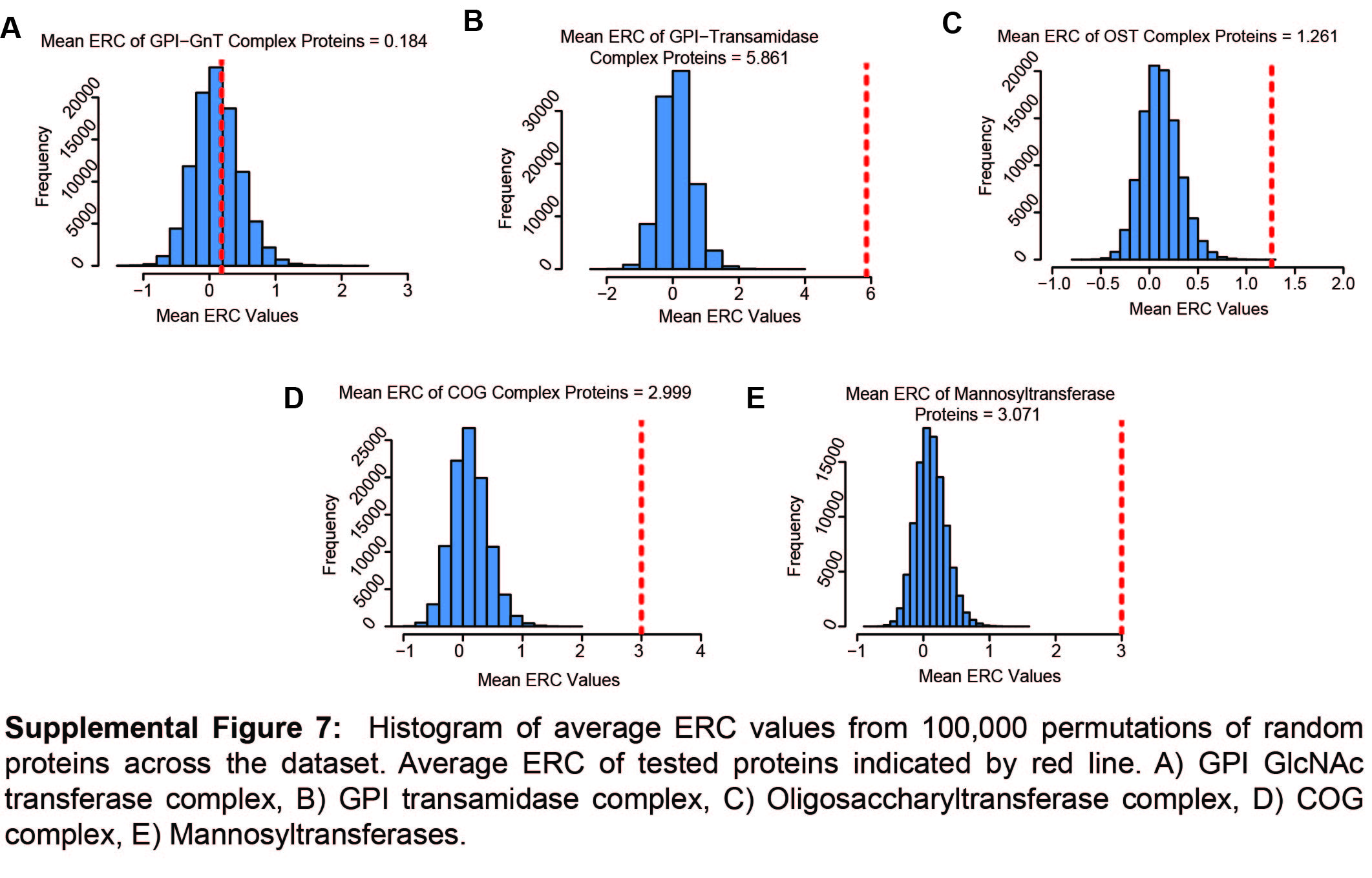

Supplement: S7 Fig — Average ERC of tested proteins indicated by red line. A) GPI GlcNAc transferase complex, B) GPI transamidase complex, C) Oligosaccharyltransferase complex, D) COG complex, E) Mannosyltransferases. (JPG) [file pgen.1011406.s007.jpg]

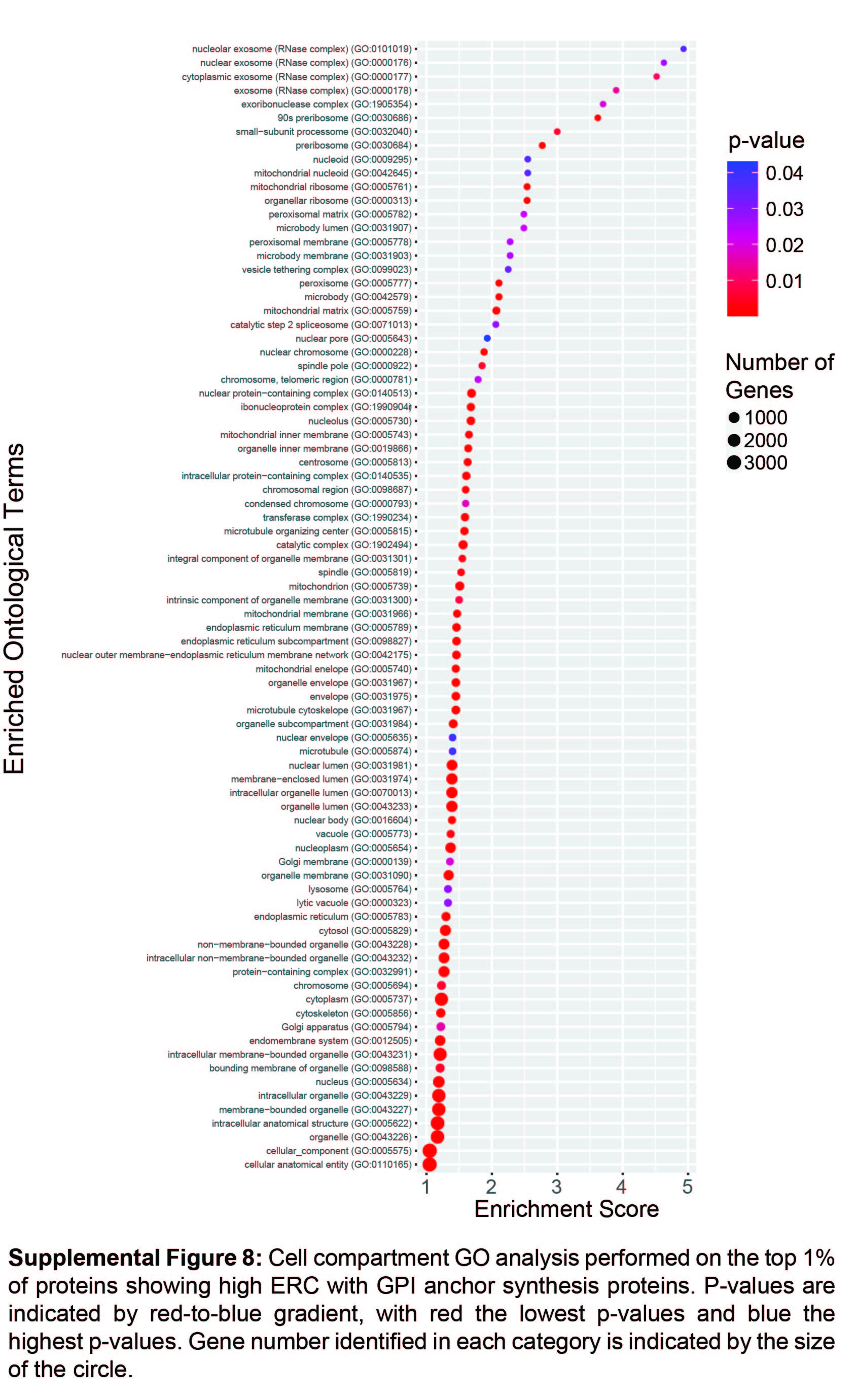

Supplement: S8 Fig — P-values are indicated by red-to-blue gradient, with red the lowest p-values and blue the highest p-values. Gene number identified in each category is indicated by the size of the circle. (JPG) [file pgen.1011406.s008.jpg]

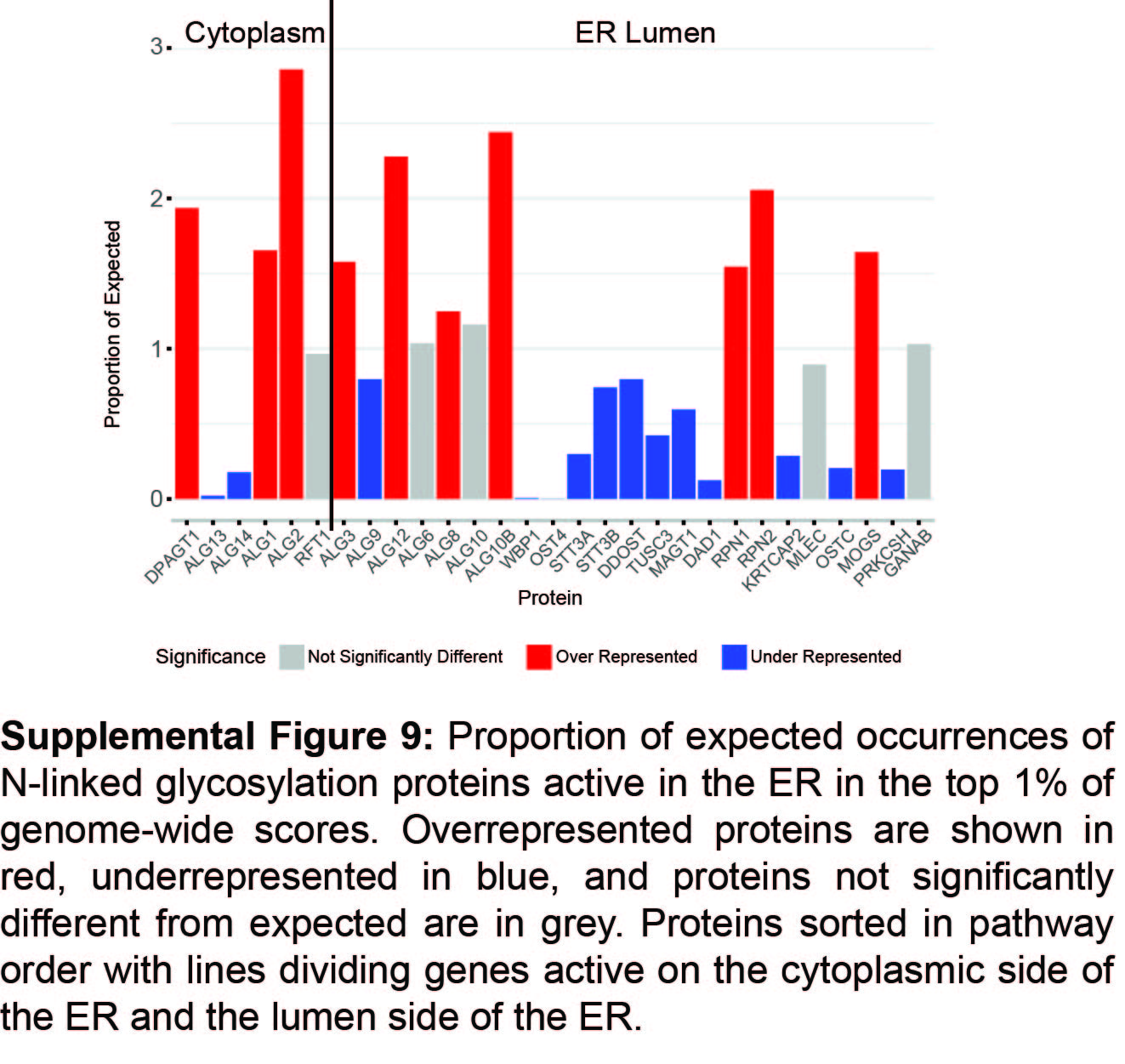

Supplement: S9 Fig — Overrepresented proteins are shown in red, underrepresented in blue, and proteins not significantly different from expected are in grey. Proteins sorted in pathway order with lines dividing genes active on the cytoplasmic side of the ER and the lumen side of the ER. (JPG) [file pgen.1011406.s009.jpg]

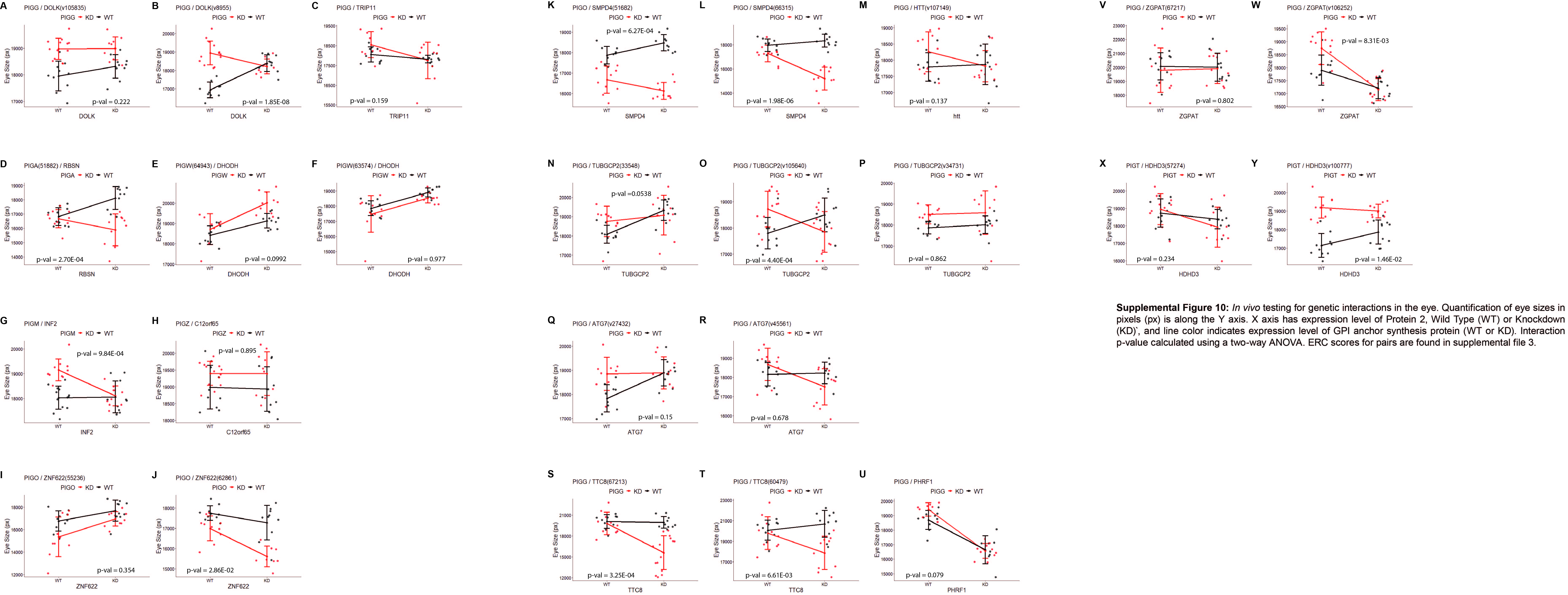

Supplement: S10 Fig — Quantification of eye sizes in pixels (px) is along the Y axis. X axis has expression level of Protein 2, Wild Type (WT) or Knockdown (KD), and line color indicates expression level of GPI anchor synthesis protein (WT or KD). Interaction p-value calculated using a two-way ANOVA. ERC scores for pairs are found in S3 Data. (JPG) [file pgen.1011406.s010.jpg]

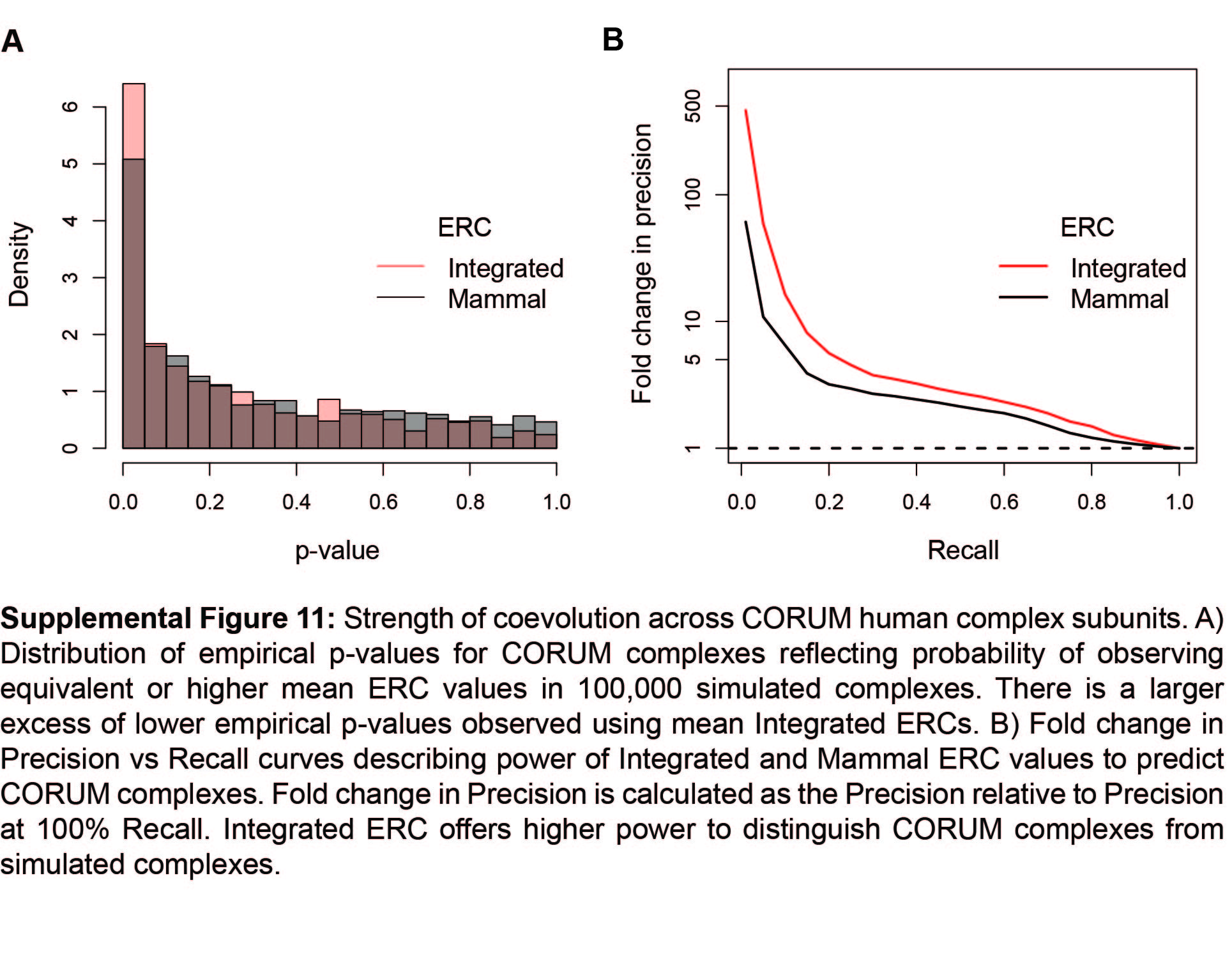

Supplement: S11 Fig — A) Distribution of empirical p-values for CORUM complexes reflecting probability of observing equivalent or higher mean ERC values in 100,000 simulated complexes. There is a larger excess of lower empirical p-values observed using mean Integrated ERCs. B) Fold change in Precision vs Recall curves describing power of Integrated and Mammal ERC values to predict CORUM complexes. Fold change in Precision is calculated as the Precision relative to Precision at 100% Recall. Integrated ERC offers higher power to distinguish CORUM complexes from simulated complexes. (JPG) [file pgen.1011406.s011.jpg]

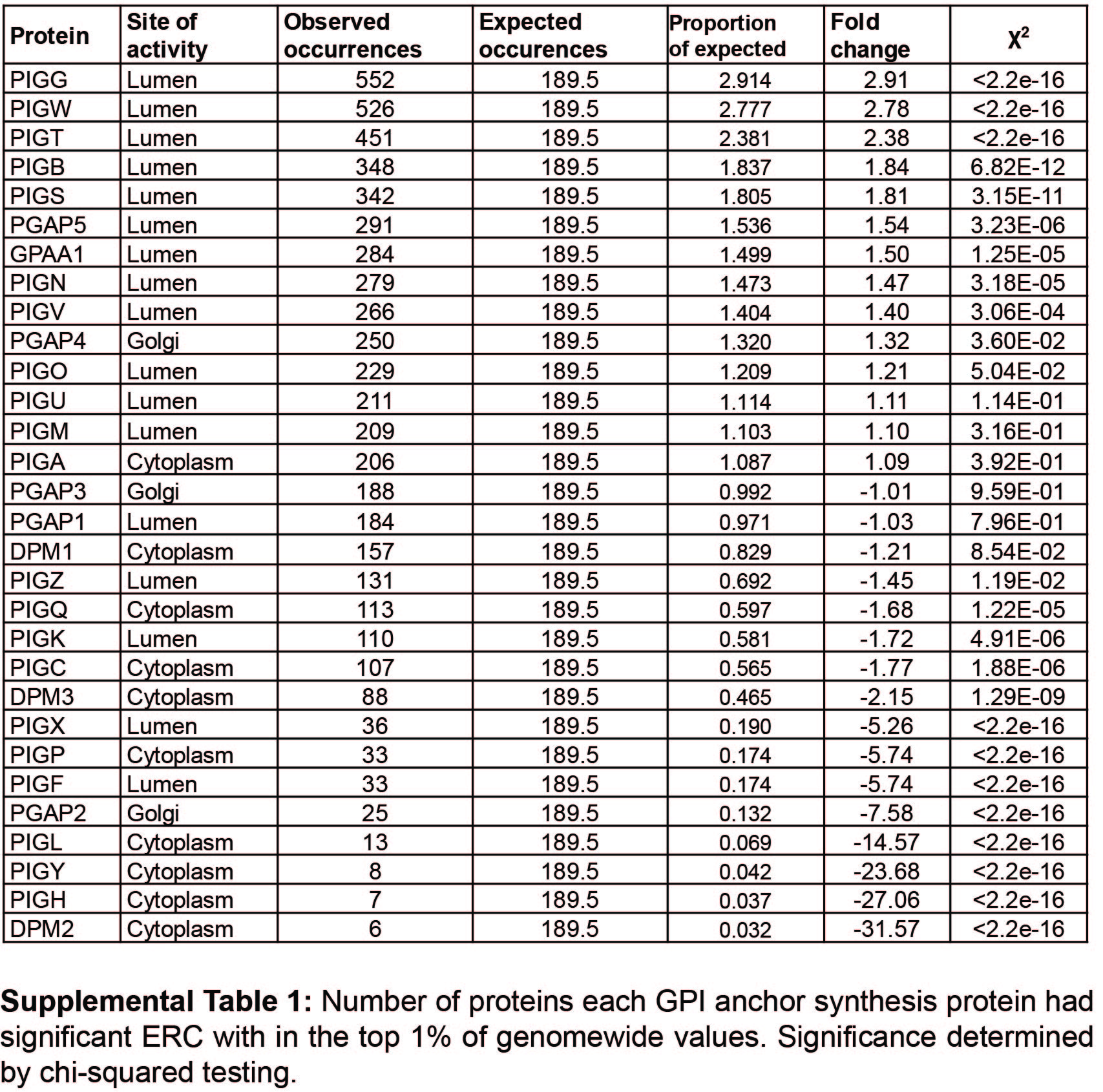

Supplement: S1 Table — Significance determined by chi-squared testing. (JPG) [file pgen.1011406.s012.jpg]
